# Identifying substance use risk based on deep neural networks and Instagram social media data

**DOI:** 10.1038/s41386-018-0247-x

**Published:** 2018-10-24

**Authors:** Saeed Hassanpour, Naofumi Tomita, Timothy DeLise, Benjamin Crosier, Lisa A. Marsch

**Affiliations:** 1 Center for Technology and Behavioral Health, Dartmouth College, Hanover, NH 03755 USA; 2 Department of Biomedical Data Science, Dartmouth College, Hanover, NH 03755 USA; 3 Department of Epidemiology, Dartmouth College, Hanover, NH 03755 USA; 4 Department of Computer Science, Dartmouth College, Hanover, NH 03755 USA; 5 Department of Psychiatry, Dartmouth College, Hanover, NH 03755 USA

**Keywords:** Risk factors, Human behaviour

## Abstract

Social media may provide new insight into our understanding of substance use and addiction. In this study, we developed a deep-learning method to automatically classify individuals’ risk for alcohol, tobacco, and drug use based on the content from their Instagram profiles. In total, 2287 active Instagram users participated in the study. Deep convolutional neural networks for images and long short-term memory (LSTM) for text were used to extract predictive features from these data for risk assessment. The evaluation of our approach on a held-out test set of 228 individuals showed that among the substances we evaluated, our method could estimate the risk of alcohol abuse with statistical significance. These results are the first to suggest that deep-learning approaches applied to social media data can be used to identify potential substance use risk behavior, such as alcohol use. Utilization of automated estimation techniques can provide new insights for the next generation of population-level risk assessment and intervention delivery.

## Introduction

Substance use is a persistent and global public health issue, affecting millions of people across every socio-demographic category. Of all deaths worldwide, 5.9% are attributable to alcohol consumption. This is greater than the proportion of deaths from HIV/AIDS, violence, and tuberculosis combined [1]. The United States is currently in the midst of a staggering drug overdose epidemic led by a surge in heroin, fentanyl, and other synthetic opioids [2, 3]. Altogether with lifestyle choices and metabolic risk factors, the use of alcohol, tobacco, and drugs are among the top ten causes of preventable deaths in the United States [4]. The misuse of prescription and illicit drugs causes over 100 deaths from overdose alone each day, dethroning motor vehicle accidents as the leading cause of injury deaths. The United States Department of Health and Human Services recently released its first-ever Surgeon General’s Report focused on addiction [5], partly spurred by the alarming national opioid epidemic [6]. The report outlines the massive social (93 million people) and economic ($442 billion) impact of illicit drug and alcohol use, yet cites a cause for optimism that is partially centered in recent research advances. These advances include innovation in information technologies—with Internet connectivity and personal computing devices now ubiquitous, there are many novel, emerging avenues for clinical research and treatment.

Behavioral health technology has demonstrated effectiveness for research, education, assessment, prevention, and treatment [7]. Web and mobile-based treatment options, called digital therapeutics, remove barriers of access to care, reduce stigma, are scalable, have high fidelity, and are inexpensive. Although healthcare systems are increasingly embracing digital behavioral health resources in their models of care, many primary-care physicians, substance use disorder treatment facilities, and emergency rooms still rely heavily on in-person assessment and treatment of substance use problems, and often do not utilize technology-based risk assessments or treatments. Additionally, among those systems that do utilize technology in their model of care, individuals in need must typically interface with such traditional healthcare systems to access these resources. A fully automated substance use screener could help facilitate more widespread adoption and could serve as a guiding example for automated screeners for other behavioral health problems.

Despite the advantages of embedding automated behavioral health risk assessments in healthcare settings, some challenges remain. For example, the alcohol, smoking, and substance involvement screening test (ASSIST) metric has admirable psychometric properties and has become a reliable tool for clinicians due to its ability to accurately describe substance use risk [8]. However, patients are often asked to recall their prior history of substance use at the time of their visit to a healthcare appointment, putting the burden of recounting many years of experience on the patient. There is already evidence that ecological momentary assessment (EMA) can reduce recall bias and increase ecological validity, indicating there are subtle environmental factors at play influencing substance use [9]. However, many current EMA techniques are cumbersome and require substantial time and effort. Accessibility of technology-based screeners, such as the one proposed in this paper, can potentially shift the burden of screening from healthcare personnel to secure methods controlled by users and/or provide an adjunct to approaches used within healthcare contexts [10].

Lifestyle choices and social networks can often influence one’s substance use risk [11, 12]. The widespread use of social media platforms provides a unique opportunity to build efficient substance use risk assessment methods. Social media images can explicitly depict substance use (e.g., consumption, inebriated behavior) [13], as well as related social (e.g., party attendance), and environmental factors (e.g., bottles, syringes, pills). It is estimated that one-third of young adults on social media have posted content depicting substance use online [13]. Social media posts thus represent potential samples of substance use risk factors and their associated social and environmental elements. Developing new, convenient, and technology-based screening methods, which utilize big data from social media based on contextual information, can advance the field beyond traditional screeners.

### Scientific premise

As of September 2017, Instagram has 800 million monthly active users [14]. Instagram is the second most popular social networking app in the United States [15]. The current massive user-base, combined with rapid rates of growth, popularity among younger users, and a highly accessible application program interface (API) make it a natural choice for our research. Social media users share information about themselves by posting image and text content, which provides data for substance use risk analysis. Social media profiles can depict sanitized versions of users, driven by self-promotion and social desirability [16]. If social media postings contain information indicative of risk, it is highly probable that this information could fuel highly accurate screening. Notably, the platform could also serve as a place of outreach, where future therapeutic services could interface with individuals who may benefit from them.

In this project, we focused on building a machine learning method that can identify high substance use risk based on social media posts on Instagram. The rich dataset from this platform combined with the recent adoption of machine learning methods in the biomedical community [17] provides a unique opportunity. Our proposed risk estimation model in this work is based on deep-learning technology, which has shown state-of-the-art results for image and text classification and analysis, and, in some cases, even exceeds human performance [18].

In recent years, deep-learning computational models, which rely on numerous levels of abstraction for data representation, are successfully deployed and used for many applications, such as autonomous mobile robots and self-driving cars [19, 20]. A major advantage of deep-learning for data analysis is eliminating the need for designing application-specific handcrafted features for training the model. The existing non-deep-learning methods for image and text analysis are based on an integrated set of features to recognize indicative patterns to accomplish the analysis. Therefore, the performance of these methods is bound by the quality of these features and the experience and skills of the designer. In contrast, because deep-learning features are automatically learned from the data as part of the training, and the model does not require an application-specific, time-consuming, handcrafted feature extraction step, training a deep-learning model can be efficient.

The technology presented in this paper represents the first prototype and proof-of-concept for a deep-learning-based substance use screening method. We expect the integration of the technology with existing substance use screening methods such as self-report surveys, interviews, and EMA can improve substance use risk assessment by capturing the social and environmental factors in social media posts that contribute to substance use risk. There is great potential to make a translational impact by harnessing deep-learning and social media data to enhance the understanding of the complex socioenvironmental factors related to substance use. In addition, this technology could be extended to create novel and efficient tools to help assess a vast array of behavioral health disorders.

## Materials and methods

### Social media data source and collection

Participants in this study were recruited primarily via the Clickworker crowdsourcing platform [21], which incentivizes participation through monetary remuneration. However, the survey, accessed via a website, was open to everyone 18 years of age or older, with some participants recruited by other means, such as word of mouth and advertisements on social media. We chose Clickworker because traditional advertising methods were less reliable and too slow for attracting participants. Other crowdsourcing platforms, such as MTurk, were not used in this study due to their specific policies on sharing social media content on these platforms.

There were three steps to the data collection process: consent, survey, and Instagram data extraction. After agreeing to the online consent form, participants were directed to a website survey interface to collect demographic and substance use information based on the National Institute on Drug Abuse’s (NIDA) Modified ASSIST substance use screener (Table S1 in Supplementary Materials) [8]. Upon completion of the survey, each participant was directed to the Instagram gateway to agree to allow our app access to their Instagram profile data. The posts were downloaded onto a secure server, if permission was granted, and stored under an anonymized unique identifier for restricted use in this study. During this step, participants needed to wait momentarily as our system recorded the information from the user’s Instagram posts. After the data had been successfully recorded, the participation was considered complete. Each participant’s data is a combination of the information they submitted through the survey (dependent variables) and the information extracted from the Instagram API (independent variables).

Out of a total of 3198 participants, we excluded 911 participants who did not have media content in Instagram to contribute to our social media-based model. The data from the remaining 2287 individuals were used to develop and evaluate the model. Also, because our proposed approach in this study is limited to analyzing text and images, we excluded video content from our dataset. The number of active Instagram users and their contributed Instagram data in our final dataset are shown in Table 1. This data collection was performed in the winter of 2016 with the approval of the Institutional review board (IRB) at Dartmouth College. Furthermore, our experimental protocols and the use of human subject data in this project were approved by the Dartmouth IRB. Data collection for this research project was conducted with informed consent from all participants and complied with the World Medical Association Declaration of Helsinki on Ethical Principles for Medical Research Involving Human Subjects.

**Table 1:** The collected Instagram data in the study

Data source	Number	Per user
Instagram user participants	2287	N/A
Collected Instagram images	466,227	201 ± 392
Collected Instagram captions	369,000	161 ± 298
Collected Instagram comments	475,000	218 ± 586

**Fig. 1:**
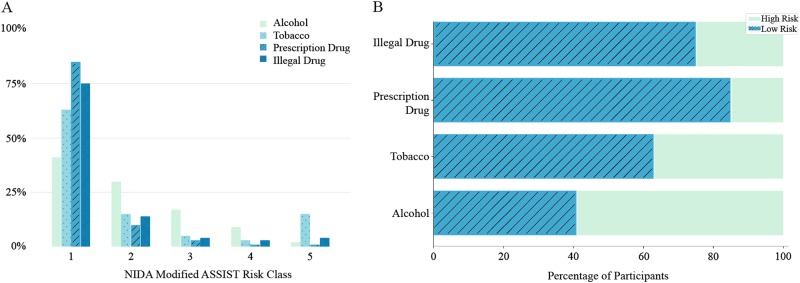
Substance use risk distribution in our dataset. **a** Substance use risk distribution among Instagram users in the dataset according to NIDA Modified ASSIST risk categories. **b** Binarized substance use risk distribution among Instagram users in the dataset

The completed NIDA Modified ASSIST substance use screeners were utilized as reference standards to label the substance use risk of individuals who participated in our study. The substance use risk distribution of the individuals in our dataset according to this survey is shown in Fig. 1a.

### Dealing with an imbalanced dataset

As is shown in Fig. 1a, the distribution of Instagram users in our dataset is substantially skewed toward the lower risk population among NIDA Modified ASSIST substance use risk classes. In this study, we converted the labels to binary classes, using the label “1” as the “low risk” class and using the rest of NIDA Modified ASSIST risk classes as the “high risk” class. This binarization is compatible with the definition of high- and low-substance risk categories according to the NIDA Modified ASSIST screener and helps with balancing the training set for developing our machine learning models. As a result, the high-risk individuals are labeled as high risk in one class rather than being scattered across multiple classes (Fig. 1b). To further improve the data balance in our model development, we oversampled from individuals in rare risk classes in our dataset in each epoch. Therefore, in each iteration of our training, the number of samples used was set to be distributed equally among low- and high-risk classes. This oversampling technique facilitated the optimization of our models.

### Features extraction from Instagram images, captions, and comments

**Fig. 2:**
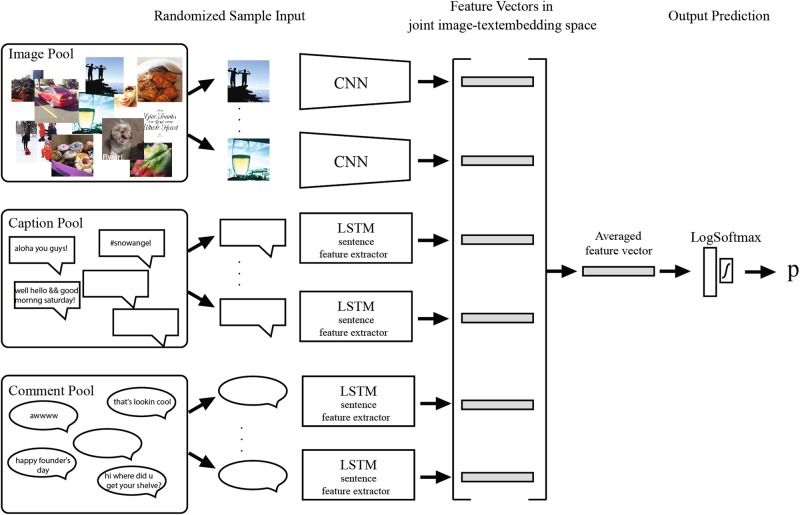
Our machine learning architecture for substance use risk estimation based on Instagram data. This architecture uses CNN and LSTM for feature extraction from images and text. A fully connected layer was trained to use the aggregated features to generate the final estimation model for substance use risk

To identify the substance use risk of Instagram users, we analyzed each user’s posts to extract indicative features from posted images, their captions, and the comments that images receive from other users. These features were extracted automatically from Instagram data through deep neural networks and were mapped into joint feature space for all images and texts from all Instagram posts. Figure 2 shows an overview of our risk estimation model.

Since image and text have different data structures and modality, different deep neural network feature extractors are required for each data type. Image features are extracted through ResNet [22], which is one of the state-of-art convolutional neural network architectures. In this study, we used a ResNet18 model, which was pre-trained on the ImageNet data repository [23]. We further fine-tuned the parameters of this model on our Instagram image dataset.

Our text feature extraction method had two parts. The first is the word2vec [24] network for generating semantic word representation. The second part is a long short-term memory or LSTM [25] network to generate semantic text representation. Word2vec is a neural network model that maps words to a semantic vector space based on word co-occurrences in a large corpus. We trained our word2vec model on the entire comments and captions from Instagram posts, in addition to the freely available repository of all English Wikipedia articles (3.6 GB of text, exported during October of 2016) on the Web [26]. Wikipedia articles were used as an additional source of word co-occurrences and sentence structures in English to enhance the distributional semantic representation outputs of our model. Our LSTM network took captions or comments as a sequence of word2vec semantic vectors per word and generated a semantic feature representation for that text.

Through this feature extraction, images, captions, and comments were mapped to the same 300-dimensional feature space for aggregation in the next step. Of note, the image and text feature extractors were jointly optimized during our training, while the word2vec model was trained on our text repository prior to training our feature extraction model.

### Aggregation of extracted features for predictive analysis

The number of posts in each Instagram profile varies drastically. For instance, the average number of posts per Instagram user in our dataset was 183.5, with a large standard deviation of 335.6. Also, the amount of data that can be analyzed in our model for each user is limited by the memory capacity of the utilized hardware. Therefore, it is necessary to develop a method that is feasible according to the memory requirements and that is applicable to all users with different numbers of posts.

To address the diversity of user data in our risk estimation model and the memory requirements, we stochastically sampled a fixed number (i.e., 20) of images, captions, and comments from an individual’s Instagram data pool and averaged the extracted feature vectors from those data to make a risk estimation. The data sampling was performed with replacement, which made it applicable to users who had a few posts.

### Risk estimation

Our risk estimation model was based on a fully connected neural network layer with softmax normalization and a cross-entropy loss function. Intuitively, this network measures the ratio of latent features associated with high risk within a user’s data to output the estimated risk class.

### Training the feature extraction and risk estimation model

Our dataset was split into a training set (80%), a validation set (10%), and a test set (10%). Our feature extraction and risk estimation model was trained for each substance risk (i.e., alcohol, tobacco, prescription drug, and illicit drug) in an end-to-end fashion. Model training (i.e., the optimization of the neural network weights) was performed on the training set, while hyper-parameter tuning (e.g., finding the optimal learning rate, momentum, regularization weight, and the number of hidden units) was performed on the validation set. The test set was only used for evaluation.

As discussed above, for stochastic sampling, we sampled 20 data samples from each user’s images, captions, and comments with replacement as the user’s data. This sampling was repeated in every training iteration. Our deep-learning-based models were trained through stochastic gradient descent optimization. In each iteration, the computed binary cross-entropy loss is backpropagated to the network to improve performance in the next iteration.

We trained the models for 300 epochs, which took 2 weeks on a high-performance computer that was equipped with an NVIDIA Titan Xp GPU, an Intel Xeon E5-1650 CPU, and 64 GB of RAM. The initial learning rate in our training was set to 1e-3 and was reduced in half when the loss function plateaued. To prevent our model from overfitting, we employed regularizations with 1e-4 weight decay and standard image augmentation techniques [27].

### Evaluation

The trained models were evaluated on Instagram posts from 228 randomly selected Instagram users in our test set (10% of users in our dataset), with users’ corresponding risk labels from their completed surveys as the reference standard. This test set was held-out separately from the training data and the development process. We applied our risk assessment models to this test set and measured standard machine learning evaluation metrics of recall (sensitivity), precision (positive predictive value), and F-measure for our models [28]. We also calculated 95% confidence intervals for all of the performance metrics [29]. Finally, we computed the area under the receiver operating characteristic curve (AUROC) for each model to assess its general predictive performance.

## Results

**Fig. 3:**
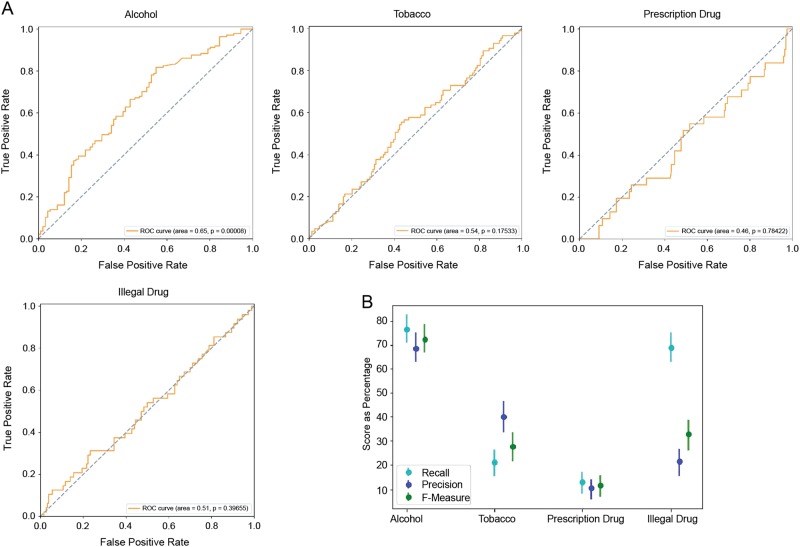
Evaluation of our approach on a held-out test set of 228 individuals. **a** Receiver operating characteristic (ROC) curves of our risk identification approach on the held-out test set for different substances. **b** The evaluation results of our machine learning model and the associated 95% confidence intervals for four different substances

The evaluation of our approach on a held-out test set of 228 individuals (10% of the 2287 total participants) showed that among four substance use risk categories, our method could identify alcohol risk based on Instagram content significantly better than that expected due to random chance. As shown in Fig. 3a, while the *p*-value of the C-statistic (i.e., AUROC) was 0.00008 for alcohol risk identification, results from our model for identifying tobacco, prescription drug, and illicit drug risk were not statistically significant. Figure 3b shows the evaluation metrics of our approach and their corresponding 95% confidence intervals for identifying alcohol risk (precision = 68.6%, recall = 76.6%, F-measure = 72.4%) and other substance risks.

### Feature analysis

To further investigate the contribution of different data sources to our alcohol risk assessment model, we performed an additional feature analysis. In this analysis, we modified the feature aggregation module of our model to exclude different data types (i.e., images, captions, and comments) for risk assessment to study their role in alcohol risk estimation. Through this experiment, we evaluated our alcohol risk assessment model in different scenarios, in which only a subset of data types was utilized. As shown in Table 2, our deep-learning model for alcohol risk estimation achieved 0.54, 0.56, and 0.60 of AUROC based on only images, captions, and comments, respectively. The AUROC of the model was 0.65 based on all three data types.

**Table 2:** The performance of different models and the contribution of different social media features and data types for alcohol use risk assessment

Model	Feature/data type	AUROC
Logistic Regression Model (Baseline)	Face features	0.54
	Alcohol-related captions	0.51
	Alcohol-related comments	0.50
	Face features and alcohol-related captions	0.54
	Face features and alcohol-related comments	0.54
	Alcohol-related captions and comments	0.51
	All three features combined	0.55
Our Deep-Learning Model	Images only	0.54
	Captions only	0.56
	Comments only	0.60
	Images and captions	0.56
	Images and comments	0.61
	Captions and comments	0.61
	All three data types combined	0.65

In addition, to show the validity of the automatically extracted features in our deep-learning approach, we developed and evaluated a logistic regression model using semi-automatically extracted features from social media text and image contents as a baseline. These semi-automatically extracted features included the appearance of alcohol-related words in captions and comments, and the number of faces in posted Instagram images. The alcohol-related terms were identified using the top 15 most semantically related words to “alcohol” according to our word2vec semantic word representation model. The number of faces in each image was automatically computed through a standard open-source face detection algorithm [30]. The training and evaluation setup for this logistic regression model was identical to the deep-learning model. In this training, we utilized grid search and cross-validation to find the optimal regularization parameters for the logistic regression model. We also tested this model on the different combinations of data types. The results of this experiment are summarized in Table 2. As shown in this table, the logistic regression model based on semi-automatically extracted features from images, captions, and comments could not achieve an AUROC better than 0.55 and the deep-learning model outperformed this baseline model by 0.10 of AUROC.

## Discussion

To the best of our knowledge, our results are the first to indicate that machine learning approaches can be used to identify potential substance use risk behavior, such as alcohol use, among social media users. We have also revealed one of the shortcomings and pitfalls of machine learning methods to deal with training on imbalanced datasets. We further this discussion below and provide a roadmap for continued research.

Recent studies have shown associations between social media and individuals’ behavioral health risks [31–40]. Particularly, a recent paper has proposed a method for analyzing photographic data from Instagram to screen for depression [35]. This is similar to our research on both the data source and also the aim of screening for behavioral health issues. The prior research posits positive results, although our project involves a significantly larger amount of data and different machine learning and risk estimation techniques. Of note, the dataset in this previous study only consisted of 166 participants and 43,950 images compared to 2287 participants and 466,227 images in our project. We have also included text analysis of comments and captions in our risk estimation model. Also, while this previous project relied on manually extracted features based on the psychology literature, we used convolutional neural networks and LSTM models for automated feature extraction, which is entirely data-driven.

Another related publication suggests that the content of social media posts is a better estimator of alcohol problems than reported alcohol consumption [41]. The study used structural equation modeling and had 364 participants who participated via an online survey. Participants responded to questions about their alcohol consumption, alcohol problems, and related social networking website usage. This pilot work highlights the social media aspect of alcohol consumption. Our research extends this insight to a broader population pool. Also, because of the automatic feature extraction from social media data in our approach, our new screening technique would be much less burdensome for use in practice.

We attribute the better performance of our approach for alcohol use risk identification, in comparison to tobacco and prescription/illegal drugs, to a more balanced distribution of alcohol risk categories among participants and the low prevalence of high-risk individuals in our cohort for non-alcohol use categories (see the dataset distribution in Fig. 1). The imbalanced dataset and low prevalence of high-risk individuals negatively impacted the efficacy of machine models, which rely on statistical patterns and features in each risk group. The imbalanced class problem is a known issue in machine learning [42], and this challenge deserves more investigation and expansion of the current techniques.

The low prevalence of high-risk individuals for tobacco and drug use in our dataset may be due to the low social acceptability of these substances. This low social acceptability could result in self-censorship in social media postings and responses to our risk assessment survey. Also, it cannot be assumed that a randomly selected population of social media users has high risk for drug or tobacco use. For example, a recent report shows that only 30% of 18 to 25-year-olds smoked tobacco in the past month [43]. The report also indicates that only 7% of the same age group suffered from an illicit drug use disorder. In contrast, alcohol is considered to be more socially acceptable, is served at most restaurants, and is prevalent at social events. The imbalance of the proportion of substance users to non-users underscores the need to improve our learning model to better handle unbalanced datasets and to target recruitment of higher substance using populations in future studies.

The work presented here illustrates an approach for alcohol use risk assessment using social media data. However, due to the low prevalence of high-risk individuals for non-alcohol use categories in our cohort, our model was not as effective for identifying substance use risk among tobacco and prescription/illicit drug users. As future work, we plan to collect additional data from individuals at high risk for tobacco and prescription/illicit drug use through collaboration with the NIDA Clinical Trials Network to examine the efficacy of our approach in using social media to identify substance use risk in such populations.

In this study, we also trained an alternative machine learning model (i.e., logistic regression) based on semi-automatically extracted features from social media text and image contents and showed the superiority of the proposed deep-learning model and its automatic feature extraction strategy for alcohol use risk assessment (Table 2). In addition, to gain further insight into the social media data characteristics that contribute to high-risk alcohol use, we performed an additional analysis to compile and compare a list of descriptive statistics for each risk group. These characteristics are summarized in Table S2 and S3 in Supplementary Materials. According to this analysis, younger individuals with more Instagram posts tend to be at a higher risk for alcohol use (i.e., *p*-value < 0.05). While the association of gender and alcohol risk was not statistically significant in our dataset, we found race is a significant factor in this domain (Table S2). As an example, according to our analysis, the odds ratio of being at high risk for alcohol use among White individuals is about 90% higher than Asian or Black individuals. We also observed that lower numbers of captions and comments per post are associated with higher alcohol risk (Table S3). In addition, a higher number of alcohol-related captions and comments are found to be indicative of high-risk alcohol use. Table S3 also shows that a higher number of faces in Instagram posts is associated with higher alcohol use risk among Instagram users, and that is aligned with previously published observations [38]. Notably, while the proposed deep-learning methodology in this paper relies directly on raw social media images and texts to infer substance use risk, these statistically strong associations indicate the utility of incorporating users’ meta-information and characteristics in substance risk estimation models, which our team will pursue in future work to improve the performance of the model.

Of note, the proposed approach in this paper is mostly focused on behavioral health predictive analysis based on features that are automatically generated by the model. A limitation of this model lies in its “black-box” nature and the lack of comprehensible features. As a result, our model can classify substance use risk, but cannot provide explicit insight into specific elements of increased substance use risk in Instagram posts. Although Table 2 indicates all three Instagram data types (i.e., images, captions, and comments) contributed to alcohol risk assessment, the automatically extracted features from these data that influence the result outcomes are not explored in this paper. In the future, we plan to leverage deep neural network visualization and interpretation approaches to highlight indicative features in Instagram images, captions, and comments for substance use risk assessment and identify the characteristics of these features that contribute to high-risk behavior. In addition, as future work, our approach can be extended to other social network platforms, such as Facebook and Twitter. We will also consider investigating and developing a similar risk assessment approach for other behavioral health disorders, such as depression, based on social media content [40].

The technology developed and evaluated in this project has great potential to make an impact by serving as a low-burden and context-sensitive screening mechanism for clinical research and treatment applications. This screening technology is well suited to be integrated into existing scientifically founded, technology-based substance use interventions. Such interventions typically rely on intensive surveys to tailor treatment options. Integrating the proposed assessment system into effective digital interventions can be a promising avenue for clinical treatment and research for the next generation of population-level, behavioral interventions.

To put this study in context, social media data and computational methods have been routinely used to identify and target individuals for different commercial purposes via various advertising campaigns. In fact, this trend has been promoted by social media platforms, such as Instagram, as the current terms of service of these platforms endorse the use of social media data for advertisement [44]. In this feasibility study, we aimed to show the utility of social media data as a resource for the next generation of population-level risk assessment and intervention delivery applications. At this point, many concerns regarding potential biases and user privacy issues need to be addressed alongside the implementation of appropriate rules and boundaries in this domain before the deployment of behavioral health intervention applications based on social media data is possible in real-world settings. We believe feasibility studies that indicate the utility of social media data for behavioral health will contribute to the ongoing discussion on deciding judicious terms and conditions for the use of social media data in applications that would benefit the health of individuals and the public overall.

## Funding and disclosure

This research was supported in part by a National Institute on Drug Abuse grant, P30DA029926, and a pilot grant from the Office of the Provost at Dartmouth College. The authors declare no competing interests.

## Electronic supplementary material

Supplementary Material

## References

[1] World Health Organization. Global status report on alcohol and health; ​WHO Press, Geneva, Switzerland 2014.

[2] Ahmad FB, Rossen LM, Spencer MR, Warner MSP. Provisional drug overdose death counts; US National Center for Health Statistics, Hyattsville, MD 2018.

[3] Salam M. The Opioid Epidemic: A crisis years in the making. New York Times; 2017.

[4] Danaei G, Ding EL, Mozaffarian D, Taylor B, Rehm JMC. The preventable causes of death in the united states: comparative risk assessment of dietary, lifestyle, and metabolic risk factors. PLoS Med. 6:1-23. 10.1371/journal.pmed.1000058 PMC2667673 19399161

[5] US Substance Abuse and Mental Health Services Administration and the Office of the Surgeon General. Facing addiction in America: The surgeon general’s report on alcohol, drugs, and health. US Department of Health and Human Services, Washington, DC. 2016. 28252892

[6] Kolodny A, Courtwright DT, Hwang CS, Kreiner P, Eadie JL, Clark TW (2015). The prescription opioid and heroin crisis: A public health approach to an epidemic of addiction. Annu Rev Public Health.

[7] Marsch LA, Lord SE, Dallery J. Behavioral healthcare and technology: using science-based innovations to transform practice. First edit. Oxford University Press; New York, NY 2014.

[8] WHO ASSIST Working Group. (2002). The Alcohol, Smoking and Substance Involvement Screening Test (ASSIST): development, reliability and feasibility. Addiction.

[9] Harris SK, Louis-Jacques J, Knight JR (2014). Screening and brief intervention for alcohol and other abuse. Adolesc Med State Art Rev.

[10] Harris SK, Knight JR (2014). Putting the screen in screening. Alcohol Res.

[11] Davies G, Elison S, Ward J, Laudet A. The role of lifestyle in perpetuating substance use disorder: the Lifestyle Balance Model. Alexandre. Substance Abuse Treatment, Prevention, and Policy, 2015;10:2, 1–8. 10.1186/1747-597X-10-2 PMC4326198 25595205

[12] Beattie MC, Longabaugh R (1997). Interpersonal factors and post-treatment drinking and subjective wellbeing. Addiction.

[13] Morgan EM, Snelson C, Elison-Bowers P (2010). Image and video disclosure of substance use on social media websites. Comput Human Behav.

[14] Etherington D. Instagram now has 800 million monthly and 500 million daily active users. Tech Crunch. 2017 https://techcrunch.com/2017/09/25/instagram-now-has-800-million-monthly-and-500-million-daily-active-users/ Accessed 17 September 2018.

[15] Verto Analytics. Most popular mobile social networking apps in the United States as of February 2018, by monthly users (in millions). Statista. www.statista.com/statistics/248074/most-popular-us-social-networking-apps-ranked-by-audience/ 2018. Accessed 17 September 2018.

[16] Ellison NB, Steinfield C, Lampe C (2007). The benefits of facebook “friends:” social capital and college students’ use of online social network sites. J Comput Commun.

[17] Park C, Took CC, Seong JK (2018). Machine learning in biomedical engineering. Biomed Eng Lett.

[18] LeCun Y, Bengio Y, Hinton G (2015). Deep learning. Nature.

[19] Farabet C, Couprie C, Najman L, LeCun Y. Scene parsing with multiscale feature learning, purity trees, and optimal covers. arXiv Prepr arXiv12022160. 2012

[20] Hadsell R, Sermanet P, Ben J, Erkan A, Scoffier M, Kavukcuoglu K (2009). Learning long-range vision for autonomous off-road driving. J F Robot Wiley Online Libr.

[21] Clickworker. https://www.clickworker.com/. Accessed 17 September 2018.

[22] He K, Zhang X, Ren S, Sun J. Deep residual learning for image recognition. 2016 IEEE Conf Comput VisPattern Recognit. 2016:770-778 10.1109/CVPR.2016.90.

[23] Russakovsky O, Deng J, Su H, Krause J, Satheesh S, Ma S (2015). ImageNet large scale visual recognition challenge. Int J Comput Vis Springe US.

[24] Mikolov T, Chen K, Corrado G, Dean J. Efficient estimation of word representations in vector space. CoRR. 2013;1–12. abs/1301.3781

[25] Hochreiter S, Schmidhuber J. Long short-term memory. Neural Comput. 1997;9:1735–1780. 10.1162/neco.1997.9.8.1735 9377276

[26] Wikimedia Foundation. Wikimedia downloads. https://dumps.wikimedia.org/. Accessed 17 September 2018.

[27] Krizhevsky A, Sutskever I, Hinton GE. ImageNet Classification with Deep Convolutional Neural Networks. In: Pereira F, Burges CJC, Bottou L, Weinberger KQ, editors. Adv Neural Inf Process Syst 25. Curran Associates, Inc.; 2012. p. 1097–105.

[28] Powers DM. Evaluation: from precision, recall and F-measure to ROC, informedness, markedness and correlation. Bioinfo Publications; Journal of Machine Learning Technologies. 2011;2:37–63.

[29] Clopper CJ, Pearson ES (1934). The use of confidence or fiducial limits illustrated in the case of the binomial. Biom JSTOR.

[30] Baltrusaitis T, Zadeh A, Lim YC, Morency L-P. OpenFace 2.0: Facial Behavior Analysis Toolkit. 2018 13th IEEE Int Conf Autom Face Gesture Recognit (FG 2018). IEEE; 2018. p. 59–66.

[31] Moreno MA, Whitehill JM (2014). Influence of social media on alcohol use in adolescents and young adults. Alcohol Res.

[32] Cabrera-Nguyen EP, Cavazos-Rehg P, Krauss M, Bierut LJ, Moreno MA (2016). Young adults’ exposure to alcohol- and marijuana-related content on twitter. J Stud Alcohol Drugs.

[33] Huang GC, Unger JB, Soto D, Fujimoto K, Pentz MA, Jordan-Marsh M (2014). Peer influences: The impact of online and offline friendship networks on adolescent smoking and alcohol use. J Adolesc Heal.

[34] Boyle SC, LaBrie JW, Froidevaux NM, Witkovic YD (2016). Different digital paths to the keg? How exposure to peers’ alcohol-related social media content influences drinking among male and female first-year college students. Addict Behav.

[35] Reece AG, Danforth MC. Instagram photos reveal predictive markers of depression. EPJ Data Sci. 2017;6.

[36] Curtis B, Giorgi S, Buffone AEK, Ungar LH, Ashford RD, Hemmons J (2018). Can Twitter be used to predict county excessive alcohol consumption rates?. PLoS One Public Libr Sci.

[37] Stoddard SA, Bauermeister JA, Gordon-Messer D, Johns M, Zimmerman MA (2012). Permissive norms and young adults’ alcohol and marijuana use: the role of online communities. J Stud Alcohol Drugs.

[38] Hendriks H, Van den Putte B, Gebhardt WA, Moreno MA (2018). Social drinking on social media: Content analysis of the social aspects of alcohol-related posts on facebook and instagram. J Med Internet Res.

[39] Erevik EK, Torsheim T, Vedaa Oslash, Andreassen CS, pallesen S (2017). Sharing of alcohol-related content on social networking sites: Frequency, content, and correlates. J Stud Alcohol Drugs.

[40] Reece AG, Reagan AJ, Lix KLM, Dodds PS, Danforth CM, Langer EJ (2017). Forecasting the onset and course of mental illness with Twitter data. Sci Rep.

[41] Thompson CK, Romo L. College students’ drinking and posting about alcohol: Forwarding a model of motivations, behaviors, and consequences. J Health Commun. Thompson CM, Romo LK. 2016;21:1–8. 10.1080/10810730.2016.1153763 27186824

[42] He H, Garcia E. Learning from imbalanced data. IEEE Transactions on Knowledge and Data Engineering. 2009;21:1263–1284.

[43] Center for Behavioral Health Statistics and Quality. Key substance use and mental health indicators in the United States: Results from the 2015 National Survey on Drug Use and Health. US Substance Abuse and Mental Health Services Administration: Rockville, MD 2016.

[44] Instagram Data Policy. 2018. https://help.instagram.com/519522125107875. Accessed 17 September 2018.

