# Supplementary material for: Identifying substance use risk based on deep neural networks and Instagram social media data
Source: Neuropsychopharmacology. 2018 Oct 24;44(3):487–94. doi: 10.1038/s41386-018-0247-x (PMC6333814; doi:10.1038/s41386-018-0247-x)
Supplement: Supplementary file 1 — Supplementary Material [file 41386_2018_247_MOESM1_ESM.doc]

Supplementary Material

**Table S1.** NIDA Modified ASSIST screener questions with numerical risk classes used for referencing standard labels for substance use risk in our study.

| NIDA *Quick Screen* Question:  **In the past year**, how often have you used the following? | (1)  Never | (2)  Once or Twice | (3)  Monthly | (4)  Weekly | (5)  Daily or Almost Daily |
| --- | --- | --- | --- | --- | --- |
| Alcohol   - For men, 5 or more drinks a day - For women, 4 or more drinks a day |  |  |  |  |  |
| Tobacco Products |  |  |  |  |  |
| Prescription Drugs for Non-Medical Reasons |  |  |  |  |  |
| Illegal Drugs |  |  |  |  |  |

**Table S2.** The odds ratio for alcohol use risk among major racial groups in our dataset. A major racial group in our dataset covers at least one percent of the individuals (> 22) who participated in our study.

| Racial Groups | Odds Ratio |
| --- | --- |
| White vs Asian | 1.88 |
| White vs Black | 1.86 |
| White vs Hispanic | 1.15 |
| Hispanic vs Asian | 1.63 |
| Hispanic vs Black | 1.61 |
| Black vs Asian | 1.01 |

**Table S3.** Characteristic features, their associated p-values with alcohol use risk according to the Chi-squared test, and mean differences between high- and low-risk groups (Δ), indicating the inclinations toward the high-risk class, in our dataset.

| Features | p-value | Δ |
| --- | --- | --- |
| Gender | 0.246 | N/A |
| Race | < 0.05 | N/A |
| Age | < 0.05 | - 1.2 |
| # Instagram posts | < 0.05 | 49.7 |
| # Likes | 0.374 | 509.0 |
| # Follows | 0.697 | 13.2 |
| # Followers | 0.543 | 28.8 |
| Average number of captions per post | < 0.05 | - 1.1 |
| Average number of comments per post | < 0.05 | - 0.4 |
| # Alcohol related comments | < 0.05 | 0.8 |
| # Alcohol related captions | < 0.05 | 0.3 |
| # Faces in Instagram posts | < 0.05 | 46.0 |
